# Coded-GFDM for Reliable Communication in Underwater Acoustic Channels

**DOI:** 10.3390/s22072639

**Published:** 2022-03-30

**Authors:** Mohsin Murad, Imran A. Tasadduq, Pablo Otero

**Affiliations:** 1 Telecommunication Engineering School, University of Malaga, 29071 Malaga, Spain; mohsin@uma.es (M.M.); pablo.otero@uma.es (P.O.); 2 Institute of Oceanic Engineering Research, University of Malaga, 29071 Malaga, Spain; 3 Department of Computer Engineering, Umm Al-Qura University, Makkah 21955, Saudi Arabia

**Keywords:** underwater acoustic, generalized frequency division multiplexing, block codes, convolutional codes

## Abstract

The performance of the coded generalized frequency division multiplexing (GFDM) transceiver has been evaluated in a shallow underwater acoustic channel (UAC). Acoustic transmission is the scheme of choice for communication in UAC since radio waves suffer from absorption and light waves scatter. Although orthogonal frequency division multiplexing (OFDM) has found its ground for multicarrier acoustic underwater communication, it suffers from high peak to average power ratio (PAPR) and out of band (OOB) emissions. We propose a coded-GFDM based multicarrier system since GFDM has a higher spectral efficiency compared to a traditional OFDM system. In doing so, we assess two block codes, namely Bose, Chaudari, and Hocquenghem (BCH) codes, Reed-Solomon (RS) codes, and several convolutional codes. We present the error performances of these codes when used with GFDM. Furthermore, we evaluate the performance of the proposed system using two equalizers: Matched Filter (MF) and Zero-Forcing (ZF). Simulation results show that among the various block coding schemes that we tested, BCH (31,6) and RS (15,3) give the best error performance. Among the convolutional codes that we tested, rate 1/4 convolutional codes give the best performance. However, the performance of BCH and RS codes is much better than the convolutional codes. Moreover, the performance of the ZF equalizer is marginally better than the MF equalizer. In conclusion, using the channel coding schemes with GFDM improves error performance manifolds thereby increasing the reliability of the GFDM system despite slightly higher complexity.

## 1. Introduction

Underwater communication plays an important role in various offshore applications including remote monitoring, fishing industry, exploration, search and rescue, surveillance, and border safety to name a few [1]. Acoustic signals have widely been used since they do not suffer from absorption and scattering the way radio waves and light waves suffer. Low speed of sound severely limits the bandwidth at which the data transmission can take place [2]. A larger delay spread causes inter-symbol-interference (ISI) and even a small change in the position of transmitter and receiver causes Doppler shifts making the channel doubly selective [3]. Multipath properties of the channel vary according to the geometry and physical conditions such as the transmitter-receiver position as well as the sea surface and boundary. With the ever-increasing demand for high data-rate multicarrier acoustic communication, OFDM has widely been studied [4,5,6,7]. It can effectively counter ISI due to a longer symbol period. However, OFDM suffers from a high PAPR and high OOB emissions which makes it less efficient [8].

Generalized frequency division multiplexing (GFDM) [9] is a non-orthogonal modulation scheme and is among the many next generation 5G contenders proposed to deal with the limitations of the current OFDM systems. Some of its advantages include higher spectral efficiency, low OOB emissions, and flexibility [10]. Each GFDM data-block is divided into subcarriers and sub-symbols, with the subcarriers filtered through a pulse shaping filter to reduce OOB emissions. GFDM is a highly flexible scheme in time-frequency distribution with its ability to collaborate with single carrier frequency domain equalization based systems and filter-bank multicarrier systems [11]. The block-based nature of GFDM allows it to utilize several OFDM techniques, such as cyclic prefix (CP) to avoid inter symbol interference. Thus, low complexity equalizers can be employed in the frequency domain to counter the effects of multipath at the receiver side [12]. As GFDM subcarriers are filtered through a variety of pulse shapes [13] the tradeoff is a lack of orthogonality making them susceptible to self-interference at the receiver. Another disadvantage of utilizing flexible waveform is the associated complexity in implementation. However, with rapidly growing computational power and complexity reduction being an active area of research, it appears to be a viable solution for future next generation multicarrier acoustic communications.

Channel coding techniques such as forward error correction codes (FEC) have long been used for reliable transmission in wireless communication systems [14,15]. The data-rate is reduced due to the coding overhead and requires an increase in bandwidth. Communication in UAC is affected by multipath fading, absorption, and frequency dependent noise. In this work, we consider block coding schemes that include Bose, Chaudhuri, and Hocquenghem (BCH) codes and Reed Solomon (RS) codes, and a few convolutional codes for a GFDM transceiver in UAC for error-rate improvements. We compare the Symbol error rate (SER) performance of various coding schemes over varying transmitter-receiver (Tx-Rx) distances. Some contributions of this paper are:

- A coded-GFDM transceiver modeled in MATLAB for a shallow underwater acoustic channel.
- SER analysis of the proposed architecture for various transmitter and receiver distances along with the comparison of coded and uncoded GFDM modulation.

The remainder of this paper is organized as follows: Section 2 presents the state-of-art and the literature review. The coding techniques and proposed architecture of a GFDM system are described in Section 3. Section 4 explains the simulation setup and presents the results while the discussion on results is done in Section 5. Finally, Section 6 provides a brief conclusion.

## 2. Literature Review

While GFDM for underwater acoustic communication has recently gotten attention, there exists very little related work. In this section, we go through some of the recent state of the art. Rajappa et al. [16] have proposed a golden coded GFDM system for 5G radio frequency (RF) communication. Space time block code based golden codes have been employed to achieve diversity gains in a MIMO configuration. A higher capacity and improved bit-error-rates are obtained outperforming an uncoded GFDM system and OFDM. In [8], when evaluating the performance of an uncoded GFDM system for a shallow underwater acoustic channel, it has been observed that while having higher spectral efficiency and flexibility, GFDM outperforms OFDM in SER. It still has a slightly higher complexity compared to a traditional OFDM system. Woods Hole Oceanographic Institution (WHOI) Micro-Modem [17] uses BCH (64,10), BCH (128,8), and Hamming (14,9) coding schemes for reliable acoustic communication underwater.

Carrick et al. [18] proposed an improved filter structure to enhance equalization and used both convolutional and low-density parity-check (LDPC) encoding schemes. Manikandan et al. [19] evaluated the performance of various forward error correction codes. The encoders are implemented using an FPGA and it is observed that LDPC has the smallest area while RS encoder has the largest. We employed BCH codes for peak-to-average power (PAPR) reduction in OFDM for UACs [20]. It was concluded that other than BER improvements, the PAPR of an OFDM system is significantly reduced when ciphered BCH codes are utilized. Park et al. [15] have assessed the forward error correction capabilities of convolutional codes of code rate 1/2 in the presence of multipath in underwater acoustic channels. The authors suggest the proposed convolutional codes for OFDM in multipath channel without equalizers in a slow-moving scenario. Authors in [21] have proposed a BCH encoded OFDM system for underwater acoustic communication. Channel information was not available. They have employed BCH (15,11) and BCH (15,7) with interleaving. BCH encoded system outperformed an uncoded OFDM system and the encoded system that didn’t use interleaving.

Anwar et al. [22] have investigated several coding schemes and waveforms for ultra-reliable and low-latency applications in the RF domain while considering both doubly-selective as well as frequency selective channels. It is observed that turbo codes outperform all other coding schemes investigated. Hebbar and Poddar [23,24] have proposed a GFDM modulated underwater acoustic transmission system. They used the Rayleigh distribution based multipath channel model along with ambient noise. They employed root raised cosine (RRC) filter for pulse shaping. For known channel state information, the results were compared against OFDM and filter bank multicarrier (FBMC) systems. Matthe et al. [12] proposed a pre-coded GFDM for high data rate RF transmission. A variety of transforms were used for different operations and a reduction in implementation complexity was achieved. None of the works mentioned above have done a thorough evaluation of the well-known and well-established block codes i.e., BCH and Reed-Solomon, and the convolutional codes with GFDM. This paper tries to fill this gap.

## 3. System Architecture

This section details the GFDM transmitter and receiver design, FEC codes, and the shallow underwater channel that we used for performing simulations.

### 3.1. Forward Error Correction Codes

FECs encoding is performed by adding a sequence of code bits to the input sequence to be transmitted [25]. This reduces the probability of any error arising due to channel impairments. Hamming distance is the number of bit positions by which the two codes are different. The hamming distance is directly proportional to the number of bits corrected at the receiver. If D is the Hamming distance, the maximum number of bits corrected are usually less than or equal to $\left(D-1\right)/2$. In block type Hamming codes, for every k bit input block size, $\left(n-k\right)$ bits are added to create a new block of size n. This coding technique is denoted by $\left(n,k\right)$. In this work, we will use two types of FECs including cyclic block (BCH & RS) codes and convolutional codes to evaluate their performance for an acoustic GFDM system.

BCH codes are considered a big category of cyclic codes. These codes work for both binary and nonbinary alphabets. Since these codes have a rich algebraic structure, their decoding is done by using efficient decoding methods. Moreover, there exist a variety of code rates and block lengths for BCH codes that are well documented as well. These codes are also considered among the best-known codes when the requirement is to use low or moderate block lengths [26].

If t represents the number of multiple bit errors, m a positive integer with m≥3 and $t\leq 2^{m-1}$, a binary BCH code is possible with the following characteristics:

- Block length $n=2^{m}-1$
- Parity check bits: n−k≤m×t
- Minimum distance D≥2t+1

While BCH codes stem from Hamming codes, unlike Hamming codes that can only correct one-bit errors, these codes can correct multi-bit errors. One of the powerful classes of cyclic codes, their code generators are well documented [27] which makes the use of various types of BCH codes a trivial job. Using the table presented in [27], BCH code generators commonly used for the construction of BCH codes can be identified for various values of n, k, and t, up to a block length of 255 [28].

Introduced in 1960 by Reed and Solomon [29], Reed-Solomon codes are considered to be the most widely used codes in communication systems as well as data storage systems. These codes in fact are nonbinary BCH codes. Symbols of RS codes are made up of m-bit sequences with m>2. For a typical RS code, n, which is the total number of code symbols in the encoded block, and k, which is the number of data symbols are given by [26]:

(1) $$\left(n,k\right)=\left(2^{m}-1,\;2^{m}-1-2t\right)$$

In short, RS codes are in fact $2^{m}$-ary BCH codes with minimum distance $D_{\min}=2t+1$ and $1<t<2^{m}-1-1$.

Given the same encoder input and output block lengths, RS codes demonstrate the largest minimum code distance when comparing the linear codes. RS codes are known to perform extremely well against burst errors [28]. Moreover, since RS codes are nonbinary, the distance between two codewords is defined as the number of symbols in which the sequences differ and for RS codes, this minimum distance is given by:

(2) $$d_{min}=n-k+1$$

**Figure 1:**
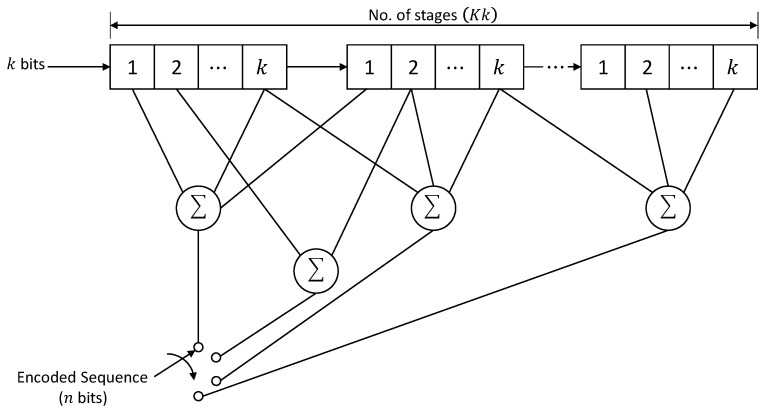
A typical convolutional encoder.

Three integers i.e., n,k, and K describe a typical convolutional code. Like the block codes, the ratio k/n is the code rate. The parameter K is called constraint length. The values of n and k are normally small integers while K controls the potential and complexity of the code. Finite-state machines are used to describe the convolutional codes. If i represents the time instant, $\sigma_{i}$ is the ith state of the encoder, and k is the information bits that enter the encoder, then the output of the encoder is n binary bits while the state of the encoder changes from $\sigma_{i-1}$ to $\sigma_{i}$. Figure 1 represents a typical convolutional encoder having shift registers of length k [26,28]. The encoder works as follows:

As a start, k bits enter the encoder at each time instance. Then the contents of the shift register are shifted to the right by k memory elements while the rightmost k bits of the register leave the encoder. During the phase when the k bits have entered the register, using modulo-2 addition, the n adders add the contents of the memory elements they are connected to. This results in the code sequence of length n.

### 3.2. GFDM Transceiver Architecture

**Figure 2:**
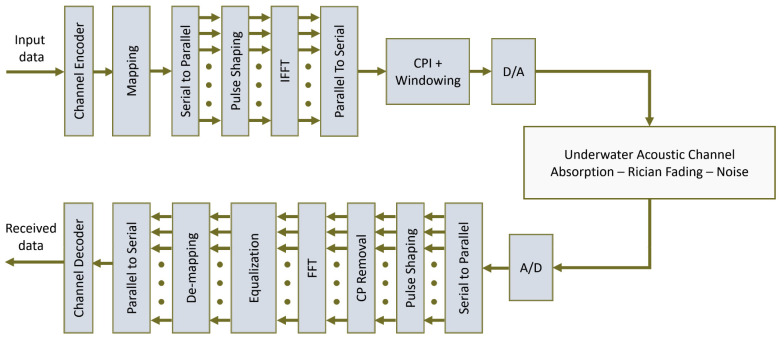
Proposed coded-GFDM transceiver architecture.

The proposed GFDM model is presented in Figure 2. Suppose the input bit stream is passed through a mapper to create multiple symbols in $2^{µ}$—QAM constellations with µ being the order of modulation. The mapped symbols are represented by $\vec{c}$. The total number of carriers used are denoted by K and M represent the sub-symbols. The serial stream is then converted to N parallel streams represented [8] by $C={\left({\vec{c}}_{0}^{T},\ldots ,{\vec{c}}_{M-1}^{T}\right)}^{T}$ where N=K×M. The output data then passes through the pulse shaping filter followed by the IFFT operation defined as:

(3) $$g_{k,m}\left[n\right]=g\left[\left(n-mK\right)\;mod\;N\right]e^{j2\pi \frac{k}{K}n}$$

It is pertinent to mention that the pulse filter expressed above as $g_{k,m}\left[n\right]$ is circularly shifted in the time domain while using a modulo operation. While several pulse-shaping filters exist in literature, we only use Raised Cosine (RC) filters in this work. The frequency response of an RC filter is given by:

(4) $$G_{RC}\left[f\right]=\frac{1}{2}\left[1-\cos\left(\pi {\mathrm{lin}}_{\alpha }\left(\frac{f}{M}\right)\right)\right]$$

The transmitted signal can be represented as follows:

(5) $$x\left[n\right]=\overset{K-1}{\underset{k=0}{\sum }}\overset{M-1}{\underset{m=0}{\sum }}g_{k,m}\left[n\right]c_{k,m}\;\;\;n=0,1,\ldots ,N-1$$

The above expression can also be written in a matrix form as follows:

(6) $$\vec{x}=A\vec{c}$$

where A [30] has dimensions of KM×KM dimensions with each vector in columns given ${\vec{g}}_{k,m}$ represented by ${\left(g_{k,m\;}\left[n\right]\right)}^{T}$ and vector $\vec{c}$ has dimensions of KM×1. Equation (5) contains A such that:

(7) $$A=\left(\;{\vec{g}}_{0,0}\ldots {\vec{g}}_{K-1,0}{\vec{g}}_{0,1}\ldots {\vec{g}}_{K-1,M-1}\right)$$

This is followed by the addition of a cyclic prefix and the signal is then converted to analog for transmission. At the receiver side, the received signal goes through an analog to digital converter, and then the cyclic prefix is removed. FFT operation is performed to obtain:

(8) $$\vec{y}=HA\vec{c}+\vec{n}$$

If the CSI is assumed to be known, we multiply each side with $H^{-1}$ and the estimated symbols $\hat{\vec{c}}$ are represented as:

(9) $$\hat{\vec{c}}=Z\tilde{\vec{y}}$$

where Z is a KM×KM matrix and its computation influences the choice of receiver design to be implemented. We consider two approaches, a matching filter (MF) receiver and a zero-forcing (ZF) receiver.

### 3.3. Shallow Underwater Acoustic Channel

The underwater acoustic channel has both time selective as well as frequency selective characteristics making it doubly selective [31]. Due to the low speed of sound, multipath and ambient noise, the bandwidth is severely limited [32]. The delay spread of the underwater channel is usually between 10 ms to 50 ms and sometimes as large as 100 ms [33]. A classic underwater channel with L time shifted paths [31] is given as:

(10) $$H\left(t,\tau \right)=\overset{L}{\underset{x=1}{\sum }}A_{x}\left(t\right)\delta \left(\tau -\tau_{x}\left(t\right)\right)$$

where $A_{x}\left(t\right)$ represents the amplitude, whereas $\tau_{x}\left(t\right)$ is xth multipath and $\delta \left(t\right)$ is the Dirac delta function. The channel envelop response can be divided into two components, the deterministic component and a random fading component [8]. This channel model is close to a real underwater channel as it contains absorption and angular pathloss, multipath fading in addition to frequency dependent noise of a realistic underwater channel. The mathematical model utilizes Rician distribution for multipath fading. For the deterministic component, the absorption is modeled as a wave propagation equation and the transfer function now becomes $H_{a}\left(f,d\right)$ [34,35] which is written as:

(11) $$H_{a}\left(f,d\right)=A_{d}e^{-\gamma \left(f\right)d}$$

where γ is the sum of absorption coefficient and phase constant while $A_{d}$ represent the scaling constant. It was observed in several studies that the multipath fading in a shallow underwater channel is better represented and modeled using Rician distribution [36,37]. We use k=2.0; m=0.4 [36] as Rician fading model parameters. The noise observed in shallow underwater acoustic channels is impulsive in nature [38] and a number of techniques have been proposed for modeling it including colored and white noise models. Comparing traditional models against the experimentally observed noise sampling reveals that a Gaussian distribution model cannot statistically represent data affected by impulsive noise whereas, stable distributions are more appropriate [39] for noise estimation purposes. Underwater ambient noise is mainly a combination of four sources including shipping activity, thermal, waves, and noise due to turbulence. The frequency dependent noise is added to the faded signal, whose power spectral density [40] is given by:

(12) $$N\left(f\right)=10\log\left(10^{\frac{N_{s}\left(f\right)}{10}}+10^{\frac{N_{tu}\left(f\right)}{10}}+10^{\frac{N_{w}\left(f\right)}{10}}+10^{\frac{N_{th}\left(f\right)}{10}}\right)$$

where $N_{s},\;N_{tu}$, $N_{w}$, and $N_{th}$ are the values associated with shipping, turbulence, wave, and thermal noise, respectively.

## 4. Simulation Setup and Results

The proposed scheme utilizes the GFDM transceiver model based on [9] along with our shallow acoustic underwater channel. Table 1 details the simulation parameters of the coding schemes and the transceiver.

A K×M binary stream is generated randomly, and the channel encoding block applies the specified encoding technique. The encoded data then undergoes M-ary mapping for a specified modulation index. In this work we have used a QAM mapper. The mapped symbols then undergo modulation according to the pulse shape selected and its roll-off factor. An IFFT operation is applied, and CP is added to every sub-symbol M.

Table 2 has the channel parameters used in MATLAB simulations. The Tx-Rx separation was varied from 500 m to 2.5 km. The Tx-Rx depth is fixed at 20 m and the maximum Doppler shift is 10 Hz. We use Matlab’s RicianChannel object for multipath fading and mathematical models are implemented for absorption and frequency dependent oceanic noise.

**Figure 3:**
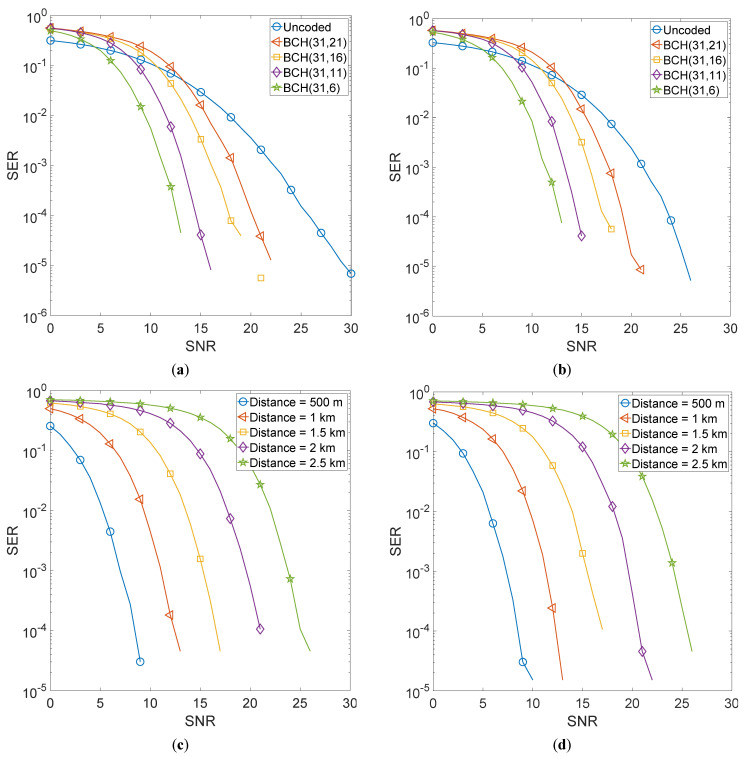
BCH encoded 4QAM-GFDM schemes. (**a**) Comparison of selected BCH schemes with MF equalizer. (**b**) Comparison of selected BCH schemes with ZF equalizer. (**c**) Error performance as a function of Tx-Rx distance for BCH (31,6) scheme with MF equalizer. (**d**) Error performance as a function of Tx-Rx distance for BCH (31,6) scheme with ZF equalizer.

Figure 3 shows the error performance of the BCH encoded GFDM scheme. Figure 3a shows a comparison of selected BCH encoding schemes, i.e., BCH (31,21), BCH (31,16), BCH (31,11), and BCH (31,6) when matched filter receiver is used for the recovery of the transmitted information and the transmitter-receiver distance is kept at 1000 m. Figure 3b shows a comparison of the same encoding schemes when a zero-forcing equalizer is used at the receiver. Figure 3c,d show the error performance of the proposed system as a function of transmitter-receiver distances. We varied this distance from 500 m to 2.5 km.

**Figure 4:**
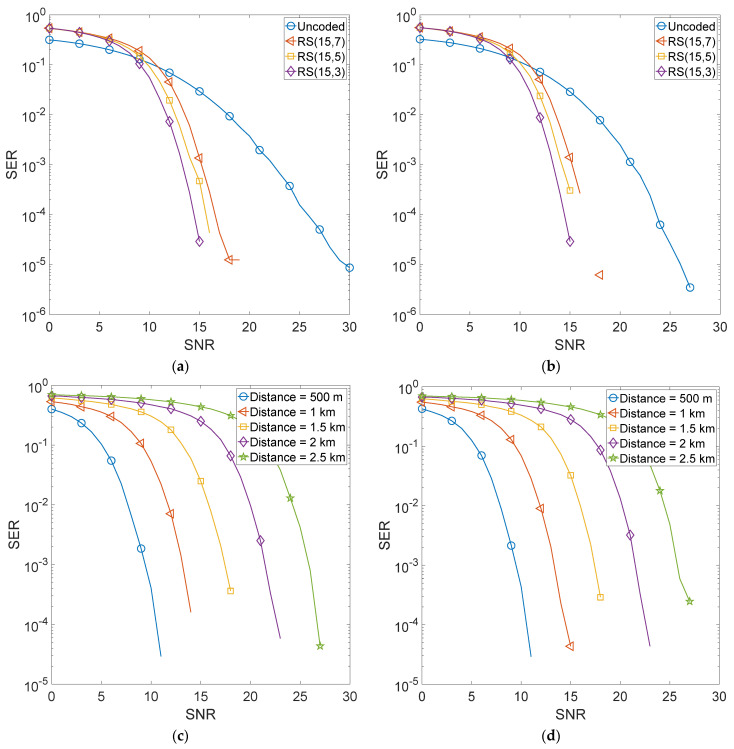
RS encoded 4QAM-GFDM schemes. (**a**) Comparison of selected RS schemes with MF equalizer. (**b**) Comparison of selected RS schemes with ZF equalizer. (**c**) Error performance as a function of Tx-Rx distance for RS (15,3) scheme with MF equalizer. (**d**) Error performance as a function of Tx-Rx distance for RS (15,3) scheme with ZF equalizer.

Figure 4 shows the error performance of the RS encoded GFDM scheme. Figure 4a shows a comparison of selected RS encoding schemes, i.e., RS (15,7), RS (15,5), and RS (15,3) when matched filter receiver is used for recovery of the transmitted information and the transmitter-receiver distance is kept at 1000 m. Figure 4b shows a comparison of the same encoding schemes when a zero-forcing equalizer is used at the receiver. Figure 4c,d show the error performance of the proposed system as a function of transmitter-receiver distances. We varied this distance from 500 m to 2.5 km.

**Figure 5:**
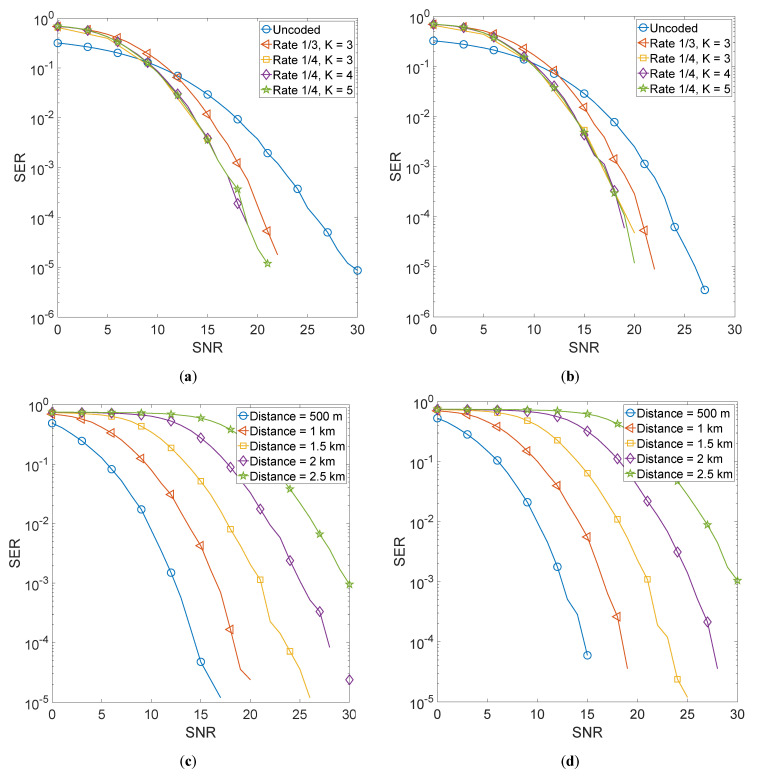
Convolutionally encoded 4QAM-GFDM schemes. (**a**) Comparison of selected convolutional codes with MF equalizer. (**b**) Comparison of selected convolutional codes with ZF equalizer. (**c**) Error performance as a function of Tx-Rx distance for rate 1/4, constraint length 5 scheme with MF equalizer. (**d**) Error performance as a function of Tx-Rx distance for rate 1/4, constraint length 5 scheme with ZF equalizer.

Figure 5 shows the error performance of four convolutionally encoded GFDM schemes. Figure 5a shows a comparison of four convolutionally encoded schemes, i.e., rate 1/3 with constraint length 3, rate 1/4 with constraint length 3, rate 1/4 with constraint length 4, and rate 1/4 with constraint length 5. The equalizer employed for the recovery of transmitted information is matched filter and the transmitter-receiver distance is kept at 1000 m. Figure 5b shows a comparison of the same encoding schemes when a zero-forcing equalizer is used at the receiver. Figure 5c,d show the error performance of the proposed system as a function of transmitter-receiver distances when a convolutional code of rate 1/4 with constraint length 5 is employed. We varied this distance from 500 m to 2.5 km.

## 5. Discussion

It is evident that using BCH encoding, improves the error performance of GFDM manifolds with BCH (31,6) giving the best performance. Moreover, the performance with a zero-forcing equalizer is marginally better than the matched filter receiver. This is because a zero-forcing scheme assumes perfect channel estimates and hence can compensate for channel impairments better than the matched filter. It is also noted that even at 2.5 km, the BCH (31,6) code gives an acceptable performance at a relatively high Signal to Noise Ratio (SNR). The performance of both matched filter and zero-forcing is quite similar and it is hard to distinguish the two plots. When using RS encoding, the error performance of GFDM improves manifolds with RS (15,3) giving the best performance. However, the difference between the error performances of the three RS schemes is relatively less than what was observed in the case of BCH encoding. Moreover, the performance with the zero-forcing equalizer is quite similar to that of the matched filter receiver and it is difficult to distinguish the two plots. Moreover, it is also observed that the use of convolutional encoding improves the error performance of GFDM but not as much as shown in the case of BCH and RS encoding. Furthermore, for both equalizers, it is hard to distinguish the performances of the three codes of rate 1/4 as they are quite similar. It is observed that at 2.5 km, high SNR is required to achieve acceptable error performance.

## 6. Conclusions

We evaluated the performance of coded-GFDM using various forward error correction codes that include BCH, RS, and convolutional codes for an acoustic GFDM transceiver in shallow underwater channels. GFDM is one of the contender waveforms for 5G and its spectral efficiency and flexibility make it an ideal OFDM alternate in underwater networks. Simulation results have shown that BCH (31,6) and RS (15,3) codes are very effective for multicarrier communication in a shallow underwater acoustic channel in terms of their error performance. The convolutional codes though substantially improve the error performance of GFDM but not as much as shown by BCH and RS. Furthermore, all the codes that we tested give exceptionally good error rates up to a transmitter receiver distance of 2.5 km. Moreover, we tested the proposed system using two different equalizers: Matched Filter (MF) and Zero-Forcing (ZF). The performance of both the equalizers was quite similar with the ZF equalizer performing marginally better than MF. This work could be extended by using more powerful codes such as Turbo codes, LDPC codes, etc., and more complex equalizers such as MMSE.

## Figures and Tables

**Table 1:** Transceiver parameters.

Parameter	Value
No. of subcarriers (K)	128
Number of time slot (M)	9
Mapping	4-QAM
Active subcarriers $\left(K_{on}\right)$	96
Active subsymbols $\left(M_{on}\right)$	7
Roll-off factor (α)	0.4
Bandwidth	10 KHz
Channel coding	BCH, RS, Convolutional
Pulse shape	Raised cosine

**Table 2:** Channel parameters.

Symbol	Quantity
TX–RX distance	500 m to 2.5 km
Depth	20 m
Max doppler shift	10 Hz
Gain vector	[0; −1.5; −2.5; −7] dB
Tau vector	[0; 1; 2; 5] ms
Atmospheric pressure	$1.01325\times 10^{5}$ Pa
Salinity	35 parts/1000
Density	10^3^ Kg/m^3^
Water temperature	25 °C

## Data Availability

Not applicable.

## References

[1] Pompili D., Akyildiz I.F. (2009). Overview of networking protocols for underwater wireless communications. IEEE Commun. Mag..

[2] Murad M., Tasadduq I.A., Otero P., Poncela J. (2021). Flexible OFDM Transceiver for Underwater Acoustic Channel: Modeling, Implementation and Parameter Tuning. Wirel. Pers. Commun..

[3] Qu F., Yang L. Basis expansion model for underwater acoustic channels?. Proceedings of the OCEANS 2008.

[4] Qiao G., Babar Z., Ma L., Liu S., Wu J. (2017). MIMO-OFDM underwater acoustic communication systems—A review. Phys. Commun..

[5] Li B., Huang J., Zhou S., Ball K., Stojanovic M., Freitag L., Willett P. Further results on high-rate MIMO-OFDM underwater acoustic communications. Proceedings of the OCEANS 2008.

[6] Tasadduq I.A., Murad M., Otero P. (2021). CPM-OFDM Performance over Underwater Acoustic Channels. J. Mar. Sci. Eng..

[7] Qasem Z.A.H., Wang J., Kuai X., Sun H., Esmaiel H. (2021). Enabling Unique Word OFDM for Underwater Acoustic Communication. IEEE Wirel. Commun. Lett..

[8] Murad M., Tasadduq I.A., Otero P. (2020). Towards Multicarrier Waveforms Beyond OFDM: Performance Analysis of GFDM Modulation for Underwater Acoustic Channels. IEEE Access.

[9] Fettweis G., Krondorf M., Bittner S. GFDM-generalized frequency division multiplexing. Proceedings of the VTC Spring 2009-IEEE 69th Vehicular Technology Conference.

[10] Li Y., Niu K., Dong C. (2019). Polar-Coded GFDM Systems. IEEE Access.

[11] Zhong J., Chen G., Mao J., Dang S., Xiao P. (2018). Iterative frequency domain equalization for MIMO-GFDM systems. IEEE Access.

[12] Matthé M., Mendes L., Gaspar I., Michailow N., Zhang D., Fettweis G.J. (2016). Precoded GFDM transceiver with low complexity time domain processing. EURASIP J. Wirel. Commun. Netw..

[13] Michailow N., Mendes L., Matthé M., Gaspar I., Festag A., Fettweis G. (2014). Robust WHT-GFDM for the next generation of wireless networks. IEEE Commun. Lett..

[14] Al-Barrak A., Al-Sherbaz A., Kanakis T., Crockett R. (2017). Enhancing BER performance limit of BCH and RS codes using multipath diversity. Computers.

[15] Park J., Seo C., Park K.-C., Yoon J.R. (2013). Effectiveness of Convolutional Code in Multipath Underwater Acoustic Channel. Jpn. J. Appl. Phys..

[16] Rajappa A.C.J., Ramadhas S.D., Anjaneyulu B.M., Kuppusamy M.R. (2020). Golden Coded GFDM for 5G Communication. Wirel. Pers. Commun..

[17] Singh S., Grund M., Bingham B., Eustice R., Singh H., Freitag L. Underwater acoustic navigation with the WHOI micro-modem. Proceedings of the OCEANS 2006.

[18] Carrick M., Reed J.H. Improved GFDM equalization in severe frequency selective fading. Proceedings of the 2017 IEEE 38th Sarnoff Symposium.

[19] Manikandan J., Manikandan M. Performance analysis of various FEC codes for wireless communication. Proceedings of the Second International Conference on Current Trends In Engineering and Technology-ICCTET 2014.

[20] Murad M., Tasadduq I.A., Otero P. (2022). Ciphered BCH Codes for PAPR Reduction in the OFDM in Underwater Acoustic Channels. J. Mar. Sci. Eng..

[21] Sabna N., Revathy R., Pillai P.S. BCH coded OFDM for undersea acoustic links. Proceedings of the 2015 International Symposium on Ocean Electronics (SYMPOL).

[22] Anwar W., Krause A., Kumar A., Franchi N., Fettweis G.P. Performance Analysis of Various Waveforms and Coding Schemes in V2X Communication Scenarios. Proceedings of the 2020 IEEE Wireless Communications and Networking Conference (WCNC).

[23] Hebbar R.P., Poddar P.G. (2020). Generalized frequency division multiplexing–based acoustic communication for underwater systems. Int. J. Commun. Syst..

[24] Hebbar R.P., Poddar P.G. Generalized frequency division multiplexing for acoustic communication in underwater systems. Proceedings of the 2017 International Conference on Circuits, Controls, and Communications (CCUBE).

[25] Clark M.P. (2000). Wireless Access Networks.

[26] Proakis J.G., Salehi M. (2008). Digital Communications.

[27] Blahut R.E. (1985). Algebraic fields, signal processing, and error control. Proc. IEEE.

[28] Sklar B. (2001). Digital Communications: Fundamentals and Applications.

[29] Reed I.S., Solomon G. (1960). Polynomial codes over certain finite fields. J. Soc. Ind. Appl. Math..

[30] Michailow N., Datta R., Krone S., Lentmaier M., Fettweis G. Generalized frequency division multiplexing: A flexible multi-carrier modulation scheme for 5th generation cellular networks. Proceedings of the German Microwave Conference (GeMiC’12).

[31] Liu C., Zakharov Y.V., Chen T. (2012). Doubly Selective Underwater Acoustic Channel Model for a Moving Transmitter/Receiver. IEEE Trans. Veh. Technol..

[32] Bocus M.J., Agrafiotis D., Doufexi A. Underwater acoustic video transmission using MIMO-FBMC. Proceedings of the 2018 OCEANS-MTS/IEEE Kobe Techno-Oceans (OTO).

[33] Wan L. (2014). Underwater Acoustic OFDM: Algorithm Design, DSP Implementation, and Field Performance. Ph.D. Dissertation.

[34] Philip M., Morse K.U.I. (1986). Theoretical Acoustics.

[35] Otero P. (2015). Fundamentos de Propagación de Ondas.

[36] Ruiz-Vega F., Clemente M.C., Otero P., Paris J.F. (2011). Ricean shadowed statistical characterization of shallow water acoustic channels for wireless communications. arXiv.

[37] Radosevic A., Proakis J.G., Stojanovic M. Statistical characterization and capacity of shallow water acoustic channels. Proceedings of the OCEANS 2009-EUROPE.

[38] Mahmood A., Chitre M. Modeling colored impulsive noise by Markov chains and alpha-stable processes. Proceedings of the OCEANS 2015—Genova.

[39] Zhang X., Ying W., Yang P., Sun M. (2020). Parameter estimation of underwater impulsive noise with the Class B model. IET Radar Sonar Navig..

[40] Stojanovic M. (2007). On the relationship between capacity and distance in an underwater acoustic communication channel. ACM SIGMOBILE Mob. Comput. Commun. Rev..

